# Temporal Considerations in Brain Metastases Radiation Therapy: The Intersection of Chronobiology and Patient Profiles

**DOI:** 10.3390/clockssleep6010014

**Published:** 2024-03-21

**Authors:** Nicolas G. Nelson, Sara E. Burke, Louis Cappelli, Lauren E. Matlack, Alexandria P. Smith, Noelle Francois, Joseph F. Lombardo, Yash B. Shah, Kuang-Yi Wen, Ayesha A. Shafi, Nicole L. Simone

**Affiliations:** 1 Department of Radiation Oncology, Sidney Kimmel Cancer Center, Thomas Jefferson University, Philadelphia, PA 19107, USA; nicnelson@g.ucla.edu (N.G.N.); sara.burke@jefferson.edu (S.E.B.); louis.cappelli@jefferson.edu (L.C.); matlac42@rowan.edu (L.E.M.); as7589@pcom.edu (A.P.S.); noelle.francois@students.jefferson.edu (N.F.); joseph.lombardo@jefferson.edu (J.F.L.);; 2 Division of Population Health, Department of Medical Oncology, Sidney Kimmel Cancer Center, Thomas Jefferson University, Philadelphia, PA 19107, USA; kuang-yi.wen@jefferson.edu; 3 Center for Prostate Disease Research, Murtha Cancer Center Research Program, Department of Surgery, Uniformed Services University of the Health Sciences, Bethesda, MD 20817, USA; ashafi@cpdr.org; 4 The Henry M. Jackson Foundation for the Advancement of Military Medicine, Inc., Bethesda, MD 20817, USA

**Keywords:** brain metastases, cancer, chronobiology, circadian clocks, radiation therapy, whole-brain radiotherapy, breast cancer, lung cancer, stress

## Abstract

The circadian system, a vital temporal regulator influencing physiological processes, has implications for cancer development and treatment response. Our study assessed circadian timing’s impact on whole-brain radiotherapy outcomes in brain metastases for personalized cancer therapy insights. The aim of the study was to evaluate circadian influence on radiation treatment timing and its correlation with clinical outcomes and to identify patient populations benefiting from interventions synchronizing circadian rhythms, considering subgroup differences and potential disparities. An IRB-approved retrospective analysis of 237 patients undergoing whole-brain radiotherapy for brain metastases (2017–2021), receiving over 80% of treatments in the morning or afternoon, was performed. Survival analyses utilized Kaplan–Meier curves. This was a single-institution study involving patients receiving whole-brain radiotherapy. Demographic, disease, and socioeconomic parameters from electronic medical records were collected. Morning treatment (*n* = 158) showed a trend toward improved overall survival vs. afternoon (*n* = 79); the median survival was 158 vs. 79 days (*p* = 0.20, HR = 0.84, CI_95%_ 0.84–0.91). Subgroup benefits for morning treatment in females (*p* = 0.04) and trends in controlled primary disease (*p* = 0.11) and breast cancer metastases (*p* = 0.08) were observed. Black patients exhibited diminished circadian influence. The present study emphasized chronobiological factors’ relevance in brain metastases radiation therapy. Morning treatment correlated with improved survival, particularly in specific subgroups. Potential circadian influence disparities were identified, laying a foundation for personalized cancer therapy and interventions synchronizing circadian rhythms for enhanced treatment efficacy.

## 1. Introduction

The circadian system is an endogenous temporal regulator that orchestrates a myriad of physiological processes in alignment with the Earth’s day–night cycle. This chronobiological mechanism is not merely a passive observer but an active participant in the homeostatic balance, influencing behavioral patterns, metabolic processes, and cellular functions. Emerging evidence suggests that circadian rhythms may play a role in the development of cancer, the process of metastasis, and the response to cancer treatments [1,2,3,4].

The concept of chronoradiotherapy has emerged from the hypothesis that the efficacy and toxicity of radiation therapy may be modulated by the timing of its administration relative to the patient’s circadian rhythms. While the body of research in this field is growing, it remains fragmented, particularly in the context of brain metastases and whole-brain radiotherapy. Studies have touched upon the potential benefits of time-of-day treatment delivery, yet the findings are heterogeneous and sometimes contradictory, underscoring a need for more focused research [5,6,7,8,9,10,11]. However, toxicity has been found to be decreased in patients treated for prostate cancer.

The literature to date has provided valuable insights but often falls short of establishing a comprehensive understanding of the temporal dynamics in radiotherapy outcomes. Notably, the majority of these studies have been limited in scope, with few addressing the circadian phase’s impact on whole-brain radiotherapy across a diverse array of primary cancers. Moreover, the existing research has tended to present isolated statistical outcomes without fully integrating them into the broader context of circadian biology’s role in cancer treatment.

Disruptions in circadian rhythms have been associated with increased cancer risk and poor prognosis, suggesting a profound underlying connection between the body’s internal clock and cancer pathophysiology. For instance, the suppression of melatonin due to circadian misalignment has been linked to carcinogenesis, particularly in shift workers exposed to irregular light cycles [12,13,14,15]. Specifically, the World Health Organization (WHO) recently designated circadian disruption as a probable carcinogen, thus raising the need to understand how biological disruption of diurnal patterns promotes tumor development and leveraging this understanding to enhance treatment with coordinated time of treatment. At the genomic level, clock genes are known to be pivotal in the DNA damage response and have been implicated in cancer development, progression, and treatment response, yet the exact pathways and interactions remain to be elucidated [16,17,18,19,20].

Our study seeks to bridge this gap by systematically evaluating the impact of circadian timing on the outcomes of whole-brain radiotherapy in patients with brain metastases. We aim to provide an understanding of how treatment timing may correlate with clinical efficacy and toxicity, potentially offering a new paradigm in the personalization of cancer therapy.

## 2. Results

Data were collected for all consecutive patients with brain metastases between 2017 and 2021. We identified a total of 2040 patients in our database who began whole-brain radiotherapy between 20 January 2017 and 2 June 2021. Treatment times were documented in the EMR for 237 patients (median survival, 91 days) who received at least 80% of their treatment in the same time window, making them eligible for the analysis. Patients were split into two groups: 158 patients who received ≥80% of their total dose before 12:00 p.m. (AM group) and 79 patients who received ≥80% of their total dose at or after 12:00 p.m. (PM group).

The population of patients who received >80% of their treatment in the AM or PM are described in Table 1. Of these, 62% were female and 50% were 65 years of age or older when diagnosed with brain metastases. The most common primary cancer site was lung (54%), followed by breast (18%). More than half of the patients were overweight or obese, as defined by BMI. Most patients had a Karnofsky performance score of ≥70. In total, for the 237 patients who received ≥80% of their total dose within a single time window, 67% had radiation mostly in the morning and 33% had >80% of their radiation in the afternoon.

**Figure 1:**
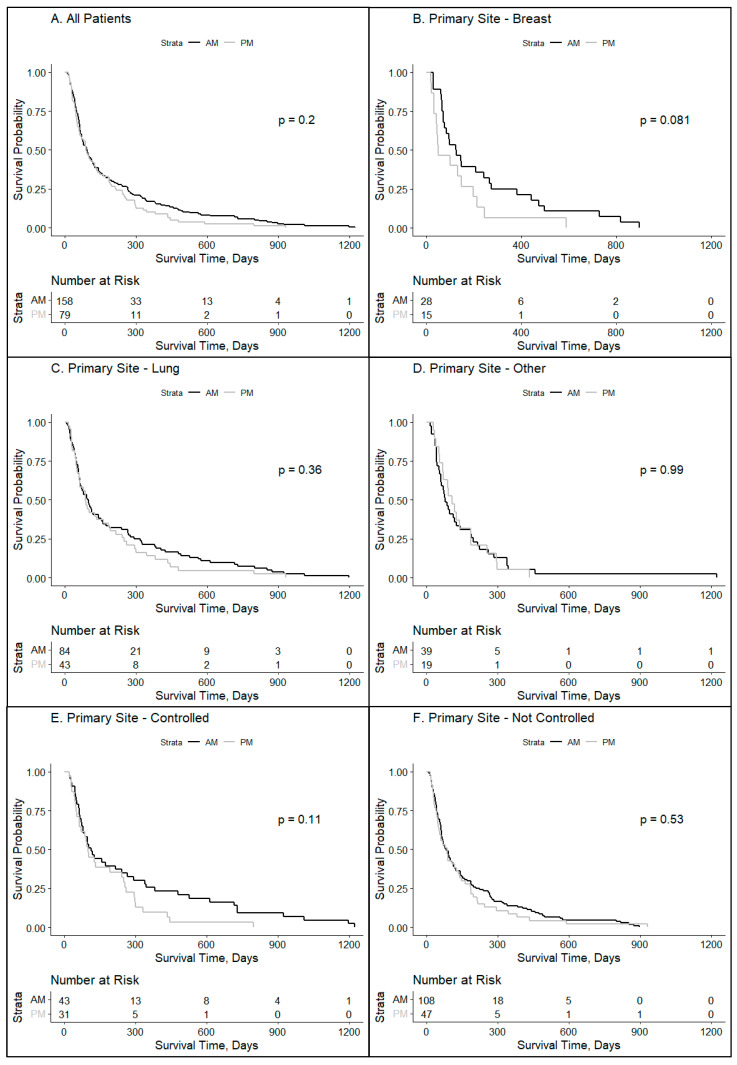
Overall impact of treatment time on brain metastases patients by disease site and disease burden. Kaplan–Meyer survival curves were used to compare groups who received ≥80% of whole-brain radiotherapy in the AM vs. ≥80% PM for specified patient subsets: (**A**) all patients (*p* = 0.20), (**B**) those with primary tumors arising from the breast (*p* = 0.08), (**C**) lung (*p* = 0.36), or (**D**) other (*p* = 0.99), (**E**) those who had their primary disease controlled (*p* = 0.11), and (**F**) those who did not have their primary disease controlled (*p* = 0.53).

Survival curves comparing the AM and PM groups for specified cohorts of patients are presented in Figure 1. For the entire cohort without subgrouping, there was a trend for improved survival for patients whose treatment was delivered in the morning (AM group) compared with the afternoon (PM group) (158 vs. 79 overall (*n* = 237, *p* = 0.20, HR = 0.84, CI_95%_ 0.84–0.91)). To assess associations of chronobiology impact on disease status, cancer subtypes were evaluated as well as the disease status for those who had controlled metastatic cancer other than in the brain or those with a more widespread disease. The breast cancer cohort revealed a trend toward survival advantage for morning radiation (*n* = 43, *p* = 0.081, HR = 0.57, CI_95%_ 0.922–3.38) with median survival in days (for AM vs. PM groups) being 124.5 vs. 50 for the breast cancer cohort, with no notable differences for the lung cancer cohort (96 vs. 87) and for other known primaries (78 vs. 108).

To determine subsets of patients who would most benefit from modulating the timing of radiation delivery or chronobiology modulation, patient characteristics, including gender, age, BMI, and KPS, were assessed to determine the impact of chronobiology on radiation outcomes. The subgroup analysis demonstrated a significant survival advantage associated with morning radiation for females (*n* = 147, *p* = 0.04, HR = 0.69, CI_95%_ 1.014–2.077), but not for males (*n* = 90, *p* = 0.55, HR = 1.14, CI_95%_ 0.57–1.35). Median survivals in days (for AM vs. PM groups) were 95.5 vs. 88 for females and 72.5 vs. 90.5 for males. We noted a trend toward longer survival after morning radiation in patients with BMI < 25 that did not reach significance (*n* = 91, *p* = 0.053).

The impact of self-defined race on chronobiology was next assessed. Race was not significantly impacted by the time of treatment. After this assessment, linear regression was performed to understand if BMI and age had a statistically significant impact on survival rate. When analyzing patients whose BMI was under 25, BMI (*p* = 0.006) was shown to negatively impact the survival rate. For every one-unit increase in BMI for patients whose BMI was under 25, the survival rate decreased by 28.36 days. Age was not shown to significantly impact the survival rate (*p* = 0.47).

## 3. Discussion

Chronotherapy has gained increasing attention for its potential in cancer care. Given the connections between chronobiology and cancer, the potential effects of chronoradiotherapy would be expected in the setting of robust circadian rhythms [1,2,3,4,6]. Our findings indicate a trend toward improved overall survival for patients receiving morning radiation, particularly evident in cohorts with controlled primary disease and those with breast cancer brain metastases. Notably, female patients also demonstrated a statistically significant survival advantage in the morning treatment group, regardless of subtyping for primary cancer. These results underscore the relevance of considering circadian timing in radiation therapy planning and suggest avenues for further investigation and potential interventions to enhance treatment efficacy.

Radiation treatment delivered in the morning trended toward a significant improvement in survival, which was more beneficial for patients having well-controlled disease or those with a breast cancer primary tumor that metastasized to the brain. Previous studies have explored the timing of radiation therapy for brain metastases, including stereotactic radiosurgery and whole-brain radiotherapy [14,15,20]. Specifically, one study demonstrated that females with brain metastases treated in the same specific window of time (08:00–11:00 a.m., 11:00 a.m.–14:00 p.m., or 14:00 h–17:00 h; *p* = 0.14) had improved survival [21]. For this study, the authors sought to expand on previous findings by comparing two distinct cohorts: one receiving at least 80% of whole-brain treatments before noon and the other at least 80% in the afternoon. The intent was to capture the broader physiologic circadian differences between the timing of the treated groups. Taken together, prior studies and this present study demonstrate a convergence of results showing the contribution of circadian rhythm impact on outcomes for metastatic patients [22]. It was not surprising that there was a trend toward a significant impact of circadian influence on brain metastases originating from breast cancer due to the strong established relationship between the impact of chronobiology as noted by night shift workers and increased breast cancer risk which was first reported in 1969 [6,7,23]. Since that time, the molecular underpinnings of the impact of chronobiology on breast cancer progression have been investigated with clock proteins, such as PER1 and PER2, having notable dysregulation in advanced cancers [24]. Our study also noted the influence of circadian regulation for patients with well-controlled disease other than brain metastases which was not seen in patients with poorly controlled disease (Figure 1E,F). When disease burden is significant, circadian misalignment decreases the influence of clock proteins on tumor control [25]. Therefore, a future strategy would be to artificially augment the circadian response with novel therapeutics to improve radiation outcomes.

**Figure 2:**
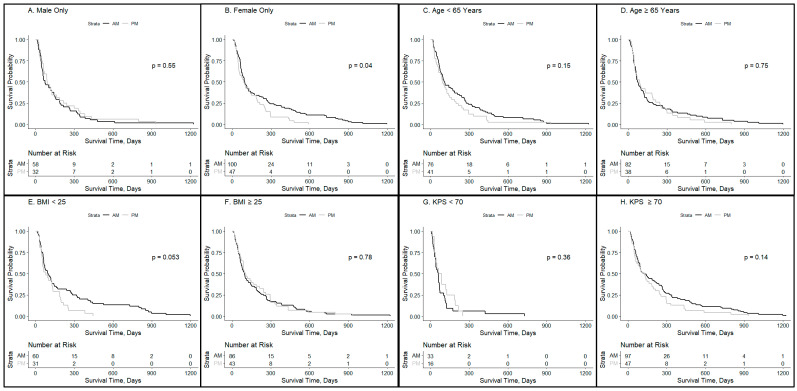
Patient characteristics impacting the timing of radiation delivery for brain metastases patients. Kaplan–Meyer survival curves were used to compare groups who received ≥80% of whole-brain radiotherapy in the AM vs. ≥80% PM for specified patient subsets: (**A**) male patients (*p* = 0.55), (**B**) female patients (*p* = 0.04), (**C**) patients who were younger than 65 years (*p* = 0.15), (**D**) patients who were 65 years or older (*p* = 0.75), (**E**) patients whose BMI was under 25 (*p* = 0.053), (**F**) patients whose BMI was over 25 (*p* = 0.78), (**G**) patients whose KPS was under 70 (*p* = 0.36), and (**H**) patients whose KPS was 70 or higher (*p* = 0.14).

In evaluating the influence of chronobiology on patient characteristics of brain metastases patients, a significant benefit was noted in female patients (Figure 2A,B), while those who were younger did not have metabolic dysfunction (as determined by BMI), had optimal performance status (KPS > 70), and had a trend toward a benefit to being treated in the morning with radiation. Our study noted a survival advantage among females with brain metastases who received whole-brain radiotherapy before 12:00 h compared with those who received treatment in the afternoon (Figure 2). The sex-dependent survival difference noted can be attributed to the interplay between circadian signaling and sex hormones [26]. Women have melatonin receptors on their ovaries that stimulate estrogen production, which in part may explain the preferential circadian influence on our female population [27]. In fact, preclinical experiments are underway using a novel melatonin–tamoxifen conjugate as an anti-cancer drug in breast cancer [28,29,30,31]. Additionally, circadian rhythms are said to be “set to an earlier hour in women than in men” [32]. This is congruent with the sex-dependent effect seen in our study. However, whether this difference stems from sex hormones, sexual dimorphism in the central circadian pacemaker, social factors, etc., is unknown and a limitation of our study is that we are unable to asses which may be driving that effect. Furthermore, it has been noted that circadian misalignment is associated with metabolic dysfunction [33]. This is congruent with our findings that older patients and those with an elevated BMI and lower functional status are less responsive to circadian influences (Figure 2C–H). Patients over 65 years old did not achieve a benefit from temporal treatment of radiation which aligns with the fact that the synchrony of circadian rhythms is known to decrease during the aging process [34]. The trend observed in the BMI < 25 subgroup of improved response with morning treatment may be because of chronobiology health. In particular, it has been shown that obese patients have a misaligned circadian pattern, with some studies demonstrating that this misalignment is more prevalent during the morning hours [35]. Therefore, gender and BMI should be considerations when designing future studies with interventions geared to targeting chronobiology. 

**Figure 3:**
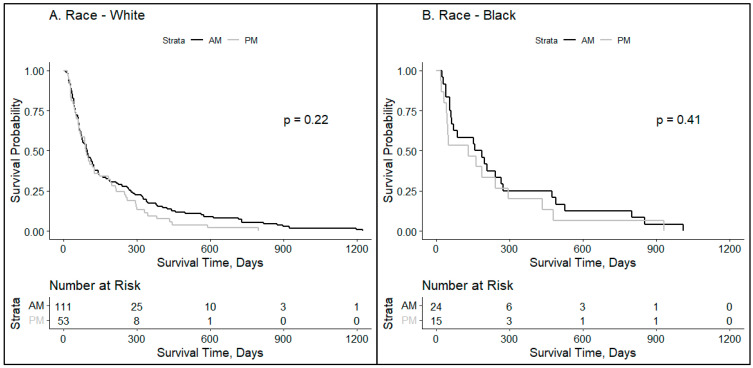
Social determinants of health impacting the timing of radiation delivery for brain metastases patients. Kaplan–Meyer survival curves were used to compare groups who received ≥80% of whole-brain radiotherapy in the AM vs. ≥80% PM for specified patient subsets: (**A**) White patients (*p* = 0.22) and (**B**) Black patients (*p* = 0.41).

Stressors, such as racism, experienced by patients disrupt the circadian patterns that are known to affect cancer [36]. The asynchrony of circadian impact on outcomes was noted in our patients with low socioeconomic means or those who experience racism (Figure 3). Conversely, high-income patients with less perceived stress and White patients not experiencing the stress of racism (who were more likely to have an intact circadian clock) had more of a benefit to morning-timed radiation. Interestingly, circadian misalignment has been noted in racial/ethnic minorities with a greater prevalence of night shift work, environmental factors (e.g., exposure to too much nighttime light and high noise pollution), and chronic conditions (e.g., diabetes and cardiovascular disease) [37]. In addition, Black patients have a shorter free-running circadian period, which is also known as tau and may contribute to the circadian misalignment [37]. Similarly, outside stressors are known to alter chronobiology. In fact, stress and stress hormones (cortisol, etc.) can cause circadian dysfunction by altering phase shifts [38]. This correlates with our finding that patients with less stress and less financial toxicity, as noted by living in a zip code with average salaries higher than the median income for Pennsylvania, are linked to more of a response to the timing of treatment.

There are limitations to our retrospective study, including the fact that the study relied on treatment time as a proxy for patients’ biological circadian phase, which can be misaligned with the actual biological circadian phase, especially in the setting context of cancer [39]. Therefore, a larger prospective study should be considered in this space for future investigators to utilize some indicator or circadian phase such as actigraphy, a biomarker, or a sleep diary.

This study highlights the impact of chronobiology in radiation outcomes for patients with brain metastases. This is the first study to identify populations of patients most likely to derive benefit from interventional trials designed to synchronize circadian rhythms to improve radiation outcomes. More effective strategies should be sought to improve radiation outcomes for patients with brain metastases by improving chronobiology for patients who are older, obese, and experience disparities and have increased tumor burden. Altering circadian patterns has the potential to increase the therapeutic efficacy of radiotherapy and this study is the first to discuss its ability to be a precision medicine approach by identifying populations most at risk of circadian dysregulation that will benefit from this approach. By understanding the importance of chronobiology on radiation outcomes, clinical trials could incorporate noninvasive methods of circadian resynchronization such as time-restricted diets and/or sleep interventions to align patients’ circadian rhythms to improve outcomes.

## 4. Materials and Methods

We performed a single-institution (Thomas Jefferson University), IRB-approved (22E.432), retrospective analysis on the overall survival of patients with secondary brain neoplasms treated with whole-brain radiotherapy. Whole brain radiation was delivered in 10 fractions to a dose of 30 Gy. Patients were eligible for our primary analysis if each of their treatment times was available in the electronic medical record (EMR) and at least 80% of their total radiation dose was received within a consistent time window, i.e., either before or after 12 p.m. For comparison, we performed a secondary analysis on patients who received 51–79% of their total radiation either before or after noon. We chose to evaluate the time as a binary concept since patient care in radiation oncology clinics are generally set up with morning and afternoon sessions with some flexibility of specific time of radiation delivery for the patients.

We utilized the EMR to collect patient characteristics recorded at the time they were diagnosed with brain metastases. Collected characteristics were sex, race that was self-reported, body mass index (BMI), residential zip code, primary disease site (lung, breast, non-lung or non-breast, or unknown/unspecified), age, presence of extracranial metastases, primary disease control, and Karnofsky performance scale (KPS) index. The latter four factors were used to assign patients to recursive partitioning analysis (RPA) classes I–III [40].

Patient survivals were calculated in days from the date of their first treatment of whole-brain radiotherapy. Dates of death were confirmed via local obituaries if unavailable in the EMR. Kaplan–Meyer survival curves and median survivals were analyzed using Prism 9 GraphPad (Version 9.3.0), with Mantel–Cox log-rank tests at a significance threshold of *p* < 0.05 and Mantel–Haenszel hazard ratio with 95% confidence intervals. Figures were generated in RStudio (Version 2023.09.1 Build 494).

## Figures and Tables

**Table 1:** Patient demographics. Patients included in our retrospective analysis received whole-brain radiotherapy for brain metastasis, given that at least 80% of their total dose was received either before 12:00 p.m. (≥80% AM) or after 12:00 p.m. (≥80% PM).

Patients	≥80% AM Treatment % (*n* = 158)	≥80% PM Treatment % (*n* = 79)	All Patients
Deceased	133 (84.18%)	71 (89.87%)	204 (86.08%)
Alive	25 (15.82%)	8 (10.13%)	33 (13.92%)
Total	158	79	237
Sex	≥80% AM	≥80% PM	All ≥ 80%
Female	100 (63.29%)	47 (59.49%)	147 (62.03%)
Male	58 (36.71%)	32 (40.51%)	90 (37.97%)
Age at Dx	≥80% AM	≥80% PM	All ≥ 80%
<65 Years	76 (48.10%)	41 (51.90%)	117 (49.37%)
≥65 Years	82 (51.90%)	38 (48.10%)	120 (50.63%)
Primary Site	≥80% AM	≥80% PM	All ≥ 80%
Breast	28 (17.72%)	15 (18.99%)	43 (18.14%)
Lung	84 (53.16%)	43 (54.43%)	127 (53.59%)
Other	39 (24.68%)	19 (24.05%)	58 (24.47%)
Unknown	7 (4.43%)	2 (2.53%)	9 (3.80%)
Primary Controlled	≥80% AM	≥80% PM	All ≥ 80%
Yes	43 (27.22%)	31 (39.24%)	74 (31.22%)
No	108 (68.35%)	47 (59.49%)	155 (65.40%)
N/A	7 (4.43%)	1 (1.27%)	8 (3.38%)
KPS Index	≥80% AM	≥80% PM	All ≥ 80%
≥70	97 (61.39%)	47 (59.49%)	144 (60.76%)
<70	33 (20.89%)	16 (20.25%)	49 (20.68%)
N/A	28 (17.72%)	16 (20.25%)	44 (18.57%)
RPA Group	≥80% AM	≥80% PM	All ≥ 80%
Class 1	17 (10.76%)	16 (20.25%)	33 (13.92%)
Class 2	80 (50.63%)	31 (39.24%)	111 (46.84%)
Class 3	32 (20.25%)	17 (21.52%)	49 (20.68%)
N/A	29 (18.35%)	15 (18.99%)	44 (18.57%)
Race/Ethnicity	≥80% AM	≥80% PM	All ≥ 80%
Asian	4 (2.53%)	1 (1.27%)	5 (2.11%)
Black	24 (15.19%)	15 (18.99%)	39 (16.46%)
Hispanic	4 (2.53%)	1 (1.27%)	5 (2.11%)
White	111 (70.25%)	53 (67.09%)	164 (69.25%)
N/A	15 (9.49%)	9 (11.39%)	24 (10.13%)
BMI	≥80% AM	≥80% PM	All ≥ 80%
<25	60 (37.97%)	31 (39.24%)	91 (38.40%)
25–30	48 (30.38%)	21 (26.58%)	69 (29.11%)
>30	38 (24.05%)	22 (27.85%)	60 (25.32%)
N/A	12 (7.59%)	5 (6.33%)	17 (7.17%)
Patient Zip Code Median Income	≥80% AM	≥80% PM	All ≥ 80%
<$57,550 K	52 (32.91%)	27 (34.18%)	79 (33.33%)
>$57,500 K	106 (67.09%)	52 (65.82%)	158 (66.67%)

## Data Availability

No new data were created or analyzed in this study. Data sharing is not applicable to this article.
